# The effect of scheduled antibody testing on treatment patterns in interferon-treated patients with multiple sclerosis

**DOI:** 10.1186/1471-2377-14-73

**Published:** 2014-04-04

**Authors:** Edward Fox, Barbara Green, Clyde Markowitz, Ronald Murray, Andrew D Goodman, Stephen J Glenski, Pippa Loupe, Jo Nita Cogburn

**Affiliations:** 1 Central Texas Neurology Consultants, 16040 Park Valley Drive, Suite 100, Round Rock, TX 78681, USA; 2 West County MS Center, Mercy Hospital, St. Louis, MO, USA; 3 MS Center, Neurology Department, University of Pennsylvania School of Medicine, Philadelphia, PA, USA; 4 Multiple Sclerosis Clinic of Colorado, Lone Tree, CO, USA; 5 Neurology Department, University of Rochester School of Medicine and Dentistry, Rochester, NY, USA; 6 Teva Pharmaceuticals, Kansas City, MO, USA

**Keywords:** Antibody testing, Interferon-β, Neutralizing antibodies, Serum binding antibodies

## Abstract

**Background:**

Many patients with relapsing-remitting multiple sclerosis (MS) treated with high-dose interferon-*β* (IFNβ) develop serum binding antibodies (BAb) and neutralizing antibodies (NAb). NAb reduces the biological activity of IFNβ, which contributes to clinical failure in these patients. We investigated whether access to antibody (Ab) test results would alter usual care of (IFNβ)-treated patients and whether BAb could predict NAb.

**Methods:**

This was a randomized, controlled, open-label, parallel-group, multicenter study in patients with multiple sclerosis. Subjects (n = 1358) were randomly assigned to Ab testing or usual care. BAb and NAb titres were measured using standard assays. Primary and secondary outcomes were the proportion of patients whose IFNβ therapy changed and the type of and reasons for therapy changes.

**Results:**

Therapy changes differed between the Ab testing and usual care arms (19.6% and 14.0%, respectively; *p* = 0 · 004). Results from Ab testing were more frequently reported as the reason for therapy change in the Ab testing arm than in the usual care arm (*p* < 0.0001). NAb and BAb positivity significantly increased the likelihood of therapy change and reduced IFNβ-associated adverse events. BAb titres were a significant predictor of NAb positivity (*p* = 0.0012). Initial BAb-positive and NAb-positive status in both study arms had a significant impact on the overall number of patients with a therapy change (*p* < 0.05).

**Conclusion:**

Access to Ab test results impacted therapy management. BAb titres can predict NAb positivity in patients on high-dose IFNβ.

## Background

Many patients with relapsing-remitting multiple sclerosis (MS) treated with high-dose interferon-*β* (IFNβ) develop serum binding antibodies (BAb) and neutralizing antibodies (NAb). Frequencies and titres of these antibodies may vary by IFNβ formulation and frequency and route of administration [1].

BAb bind to IFNβ but do not necessarily inhibit its biological activity, and they can be detected within the first month of therapy [2]. Approximately 50% of patients who develop BAb also develop NAb [3], and most patients who become NAb-positive (+) do so within 2 years of starting IFNβ treatment [2]. More than 40% of patients treated with high-dose preparations of IFN develop NAb [1], which reduce the biological activity of IFNβ, thus contributing to clinical failure [2,4]. Indeed, NAb(+) patients tend to have a higher annual relapse rate and an increase in active lesions as measured by magnetic resonance imaging (MRI) [2,5-11]. The appearance of NAb(+) titres precedes these outcomes [2].

The methodology to detect NAb is cumbersome and non-standardized; thus, simpler BAb assays are preferred for screening before analyzing for NAb. BAb screening has low false-negative rates and high sensitivity/specificity. There have been opposing assessments of the importance of BAb and NAb testing relative to clinical management of IFN-treated patients, specifically in Europe (European Federation of Neurologic Societies [EFNS]), America (American Academy of Neurology), and Canada [3,8,12].

This study investigated whether antibody (Ab) testing and knowledge of Ab status affect the usual care of patients treated with high-dose IFNβ. Moreover, because some guidelines (e.g., EFNS) advocate the use of BAb as a preliminary test, we explored whether BAb alone would be a potential guide for managing patients on IFNβ. Specifically, we analyzed the correlation between BAb and NAb titres and the presence of BAb as a predictor for NAb.

## Methods

### Study design

A randomized, controlled, open-label, parallel-group, multicenter observational study (registration: NCT00336557) in patients with MS was conducted at 147 centers in the United States. The study followed the principles of the Declaration of Helsinki International Conference on Harmonisation guidelines on good clinical practices and all applicable laws and regulations.

Investigators or designees explained the study procedures, risks, and potential benefits, if any, to all patients. Patients reviewed the study instructions and informed consent form and were given the time and opportunity to have any questions concerning the conduct of the study answered to their satisfaction.

The primary outcome measure was the evaluation of differences in therapy/management between clinicians who were provided NAb data and those who were not during the 12-month follow-up period. The secondary outcome measure was an assessment of the type of and reasons for changes in IFN therapy/management. Exploratory outcome measures included the relationship between BAb and NAb results, therapy/management changes, and targeted events.

Patients (N = 1358) on subcutaneously administered high-dose IFN therapy—IFNβ-1b (250 μg on alternate days) and IFNβ-1a (22 or 44 μg three-times weekly [t.i.w.])—were enrolled and randomly assigned to either Ab testing or usual care. Patients in the scheduled Ab testing arm had four study visits, at least two BAb and NAb tests over 12 months, and a final visit at 12 months. Those who had not completed 24 months of continuous therapy on the same IFN were offered an optional BAb +/− NAb at the final visit. Subjects in the usual care arm were followed for 12 months under usual care conditions with BAb and NAb testing at the initial visit and optional testing at the final visit. Additional visits during the year were at the discretion of the clinicians and patients. Investigators were informed of Ab test results for patients in the Ab testing arm only.

All patients who underwent a blood draw for Ab testing at the initial and final clinic visit in either arm were included in the exploratory analysis. Unscheduled visits and blood draws were allowed in either arm at any point during the 12-month study period.

### Patients

Patients enrolled were men or women aged ≥18 years who had a diagnosis of MS and were on high-dose IFN therapy (dosed according to manufacturer labelling) for 12 months to ≤4 years, with no more than 60 days of cumulative planned interruption of treatment. Patients were excluded if they had received oral or parenteral corticosteroid therapy within the 2 weeks before the initial visit; had been treated with immunoglobulin G (IgG) or plasmapheresis within the previous 6 months; were being treated with once-weekly intramuscular IFNβ-1a, glatiramer acetate, or any immunosuppressant; or had been previously tested for NAb.

### Outcome measures

Blood was drawn 48 hours after the last administration of IFNβ and at least 2 weeks after any use of systemic corticosteroids and sent to the Central Laboratory (FOCUS Bio-Inova, Cypress, CA) for analysis.

#### BAb assessment

BAb assessments were conducted with a capture enzyme-linked immunosorbent assay methodology to assess the presence of IgG antibodies that bind IFNβ. A sample was considered BAb(+) if the result was ≥4 · 0 units.

#### NAb assessment

Only the samples of patients who tested positive for BAb were analyzed for NAb positivity using the viral cytopathic effect assay method. Per laboratory standards, NAb values <20 units were considered negative (NAb[−]), and values ≥20 units were considered positive (NAb[+]).

Physicians were notified of NAb results for BAb(+) patients in the Ab testing group only. All physicians and patients were notified of NAb and BAb status at the conclusion of the trial. BAb-negative (BAb[−]) samples were randomly assessed for NAb to ensure quality control.

#### Management/therapy change questionnaire

The investigator evaluated the patient for a change in clinical status and recorded on a questionnaire whether or not a change in management/therapy had occurred or was planned. Factors that had an impact on the decision to change management/therapy were also recorded.

#### Safety measures

The most common adverse events (AEs) known to be associated with high-dose IFN treatment were collected at each visit (targeted events): flu-like symptoms, local injection-site reactions (red, warm to the touch, painful, raised area around site), depression, seizures, menstrual irregularities, abnormal liver function tests (alanine aminotransferase and aspartate aminotransferase), cytopenias (lymphopenia, neutropenia, leukopenia, and thrombocytopenia), and decreased hemoglobin.

### Statistical analysis

Patients were randomly assigned using a 1:1 assignment ratio and stratified by center using SAS^®^ (SAS Institute Inc., Cary, NC: version 9.1) random number procedure. The sample size of 1350 patients provided 99.6% power for a therapy change rate of 4.4% in the usual care arm and an 11.2% therapy change rate in the Ab testing arm. It also provided 61% power to demonstrate a significant difference, at an experiment-wise two-tailed α-level of 0.05 between a therapy change rate of 5% in the usual care arm and 8% in the Ab testing arm.

The modified intention-to-treat (mITT) population was used for all analyses (usual care arm: at least one post-baseline assessment of change in therapy; Ab testing arm: one BAb/NAb test that preceded an assessment of change in therapy). All analyses were conducted with SAS.

Chi-squared and Fisher’s exact tests were used to evaluate the association between the initial Ab status and the type and reasons for therapy changes, as well as the occurrence of targeted AEs. The *p* value for the outcome measures was based on the difference between study arms from a multivariate logistic regression model with the following covariates: type of MS, time from the onset of MS symptoms, type of current IFNβ treatment, and duration of current IFNβ treatment. The analysis model was adjusted for center size and decider status (multiple versus single). A *p* value <0.05 was deemed significant.

Multivariate models with repeated measures utilised all data from BAb/NAb testing and types of therapy change, reasons for therapy change, or AEs assessed at the same visit. Models were adjusted for age, sex, study arm, MS type, current IFN, time from onset of symptoms, and center size. Some parameters were not estimable because of low sample size (therapy change occurred less frequently). The linear trend *p* value tested for the presence of a linear trend across BAb/NAb groups.

Percentages of BAb(+) patients within NAb titre level were calculated at the initial visit using Ab status as a four-level categorical variable: (i) BAb(−), (ii) BAb(+)/NAb(−), (iii) BAb(+)/NAb(+) moderate (Ab titre ≥20 to <100 units, and (iv) BAb(+)/NAb(+) high (Ab titre ≥100 units).

Cross tabulations were generated showing the number of patients within each category at the initial and final study visits. McNemar’s test was used to test the equality of marginal frequencies (BAb[−] versus all combined BAb[+] results).

Receiver-operating characteristic (ROC) analysis was plotted, with each point indicating sensitivity and specificity for a particular BAb value in predicting NAb positivity [13], and the predictive ability was estimated by calculating the area under the curve (AUC).

A linear regression model and a Kendall’s tau were used to analyze the relationship between BAb and NAb. The R^2^ represented the amount of data variability that could be explained by the model and was similar to a correlation coefficient.

## Results

**Figure 1:**
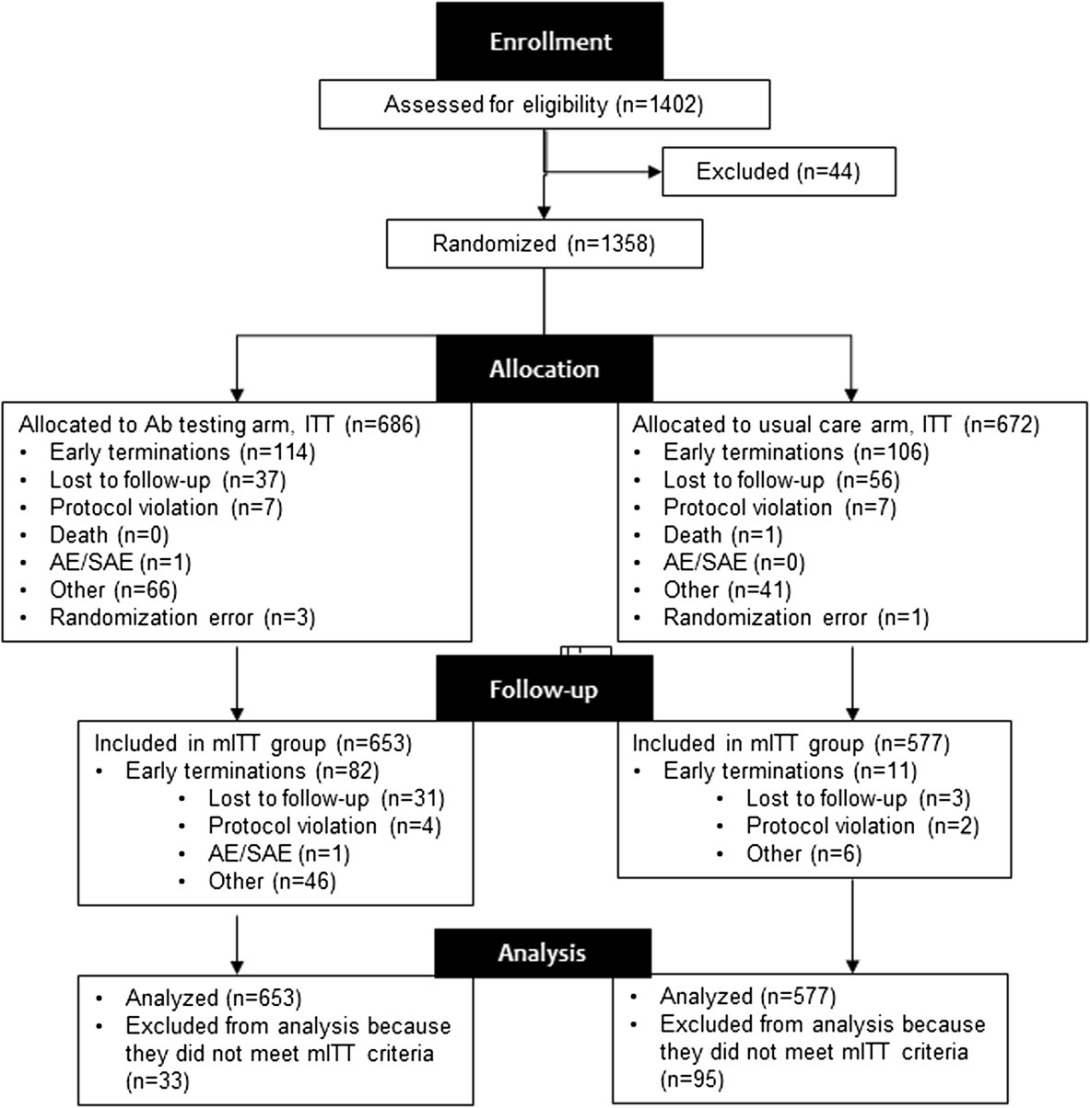
**Patient disposition.** Ab, antibody; AE, adverse event; ITT, intention-to-treat; mITT, modified ITT; SAE, serious adverse event.

The first patient was enrolled in July 2006, and the last assessment was made in April 2009. Disposition of patients is shown in Figure 1. A total of 1230 patients composed the mITT population. (One patient was excluded from the post-hoc analyses because of the absence of valid laboratory data at Visit 1.)

Table 1 shows the demographic and clinical characteristics of patients at the initial assessment stratified by study arm. At initial assessment, 18.9% of the mITT patients were NAb(+). Of the patients who were BAb(+), 46.9% were also NAb(+) at the initial assessment. No relationship was observed between initial antibody status and age, sex, or duration of illness (data not shown).

**Table 1:** Demographic and clinical characteristics of modified intention-to-treat cohort at initial assessment stratified by study arm

**Characteristic**	**Scheduled Ab testing**	**Usual care**	**All patients**
Patients, n (%)*	653 (53.1)	577 (46.9)	1230 (100)
Age in years, mean ± SD	45.3 ± 10.6	45 · 1 ± 9.8	45.2 ± 10.2
Caucasians, n (%)^†^	551 (84.4)	495 (85.8)	1046 (85.0)
Females, n (%)^†^	499 (76.4)	441 (76.4)	940 (76.4)
Years from onset of symptoms, mean ± SD	8.5 ± 7.7	8 · 5 ± 7.8	8.5 ± 7.8
**Initial high-dose IFNβ, n (%)**^**†**^			
IFNβ-1a	467 (71.5)	411 (71.2)	878 (71.4)
IFNβ-1b	186 (28.5)	166 (28.8)	352 (28.6)
**Time on IFNβ in years, mean**			
IFNβ-1a	2.2	2.3	2.3
IFNβ-1b	2.3	2.2	2.3
**Initial antibody status, n (%)**^‡^			
BAb (+)	264 (40.4)	231 (40.1)	495 (40.3)
Bab (+) NAb (+)	118 (18.1)	114 (19.8)	232 (18.9)
**NAb titre in NAb (+) patients, n (%)**			
Moderate	39 (6.0)	43 (7.5)	82 (6.7)
High	79 (12.1)	71 (12.3)	150 (12.2)

*Percentage of mITT cohort.

^†^Percentage of study arm.

^‡^Total N = 1229. Analysis excludes one patient who had no valid laboratory result at Visit 1.

Ab, antibody; BAb, serum binding antibodies; IFNβ, interferon beta; NAb, serum neutralizing antibodies; SD, standard deviation.

Subjects who received IFNβ-1a 44 μg t.i.w. had a higher proportion of positive NAb test results in both the NAb testing (14.2% [93/653]) and usual care (11.8% [68/577]) arms than patients receiving IFNβ-1b 250 μg on alternate days (5.2% [34/653] and 7.3% [42/577], respectively) or IFNβ-1a 22 μg t.i.w. (0.5% [3/653] and 0.9% [5/577], respectively).

### Primary and secondary outcome measures

#### Types of and reasons for therapy change in the Ab testing versus usual care arm

The proportion of patients who had a change in IFNβ therapy or management during the 12-month follow-up was significantly higher in the Ab testing arm than in the usual care arm (*p* = 0.004; Table 2).

**Table 2:** Types of management changes and reasons for changes by arm for modified intention-to-treat cohort

**Category**	**Scheduled Ab testing (n = 653)**	**Usual care (n = 577)**	***p*-value**
Number of patients who had a therapy change, n (%)	128 (19.6)	81 (14.0)	0.004
Type of therapy change, n (%)*^,**†**^			
Start glatiramer acetate	47 (7.2)	17 (2.9)	0.002
≥1 courses of corticosteroids for relapse	67 (10.3)	27 (4.7)	0.001
**Reasons for therapy/management change, n (%)**^**‡,§**^			
NAb result	45 (6.9)	3 (0.5)	<0.0001
Clinical composite^¶^	144 (22.1)	96 (16.6)	0.011
Other	35 (5.4)	16 (2.8)	0.011

*Patients reporting a change in type of therapy one or more times were counted once at the earliest occurrence for the same category.

^†^Types of therapy change that were not significantly different between arms included: stop or increase or decrease current interferon beta (IFNβ), start new IFNβ, start other immunotherapy, change symptomatic therapy, and closer vigilance.

^‡^Multiple reasons could be reported for one therapy change; however, patients reporting the same reason more than once were only counted once at the earliest occurrence.

^§^Reasons for therapy change that were not significantly different between arms included: clinical worsening of multiple sclerosis, magnetic resonance imaging change, side effect, other concurrent illness/adverse event, laboratory abnormality, and administrative/logistical reason.

^¶^Either clinical worsening or change on magnetic resonance imaging.

Ab, antibody; NAb, serum neutralizing antibody.

The main therapy/management changes for both arms are shown in Table 2. Significantly more patients in the Ab testing arm initiated glatiramer acetate or ≥1 course(s) of corticosteroids for relapse than in the usual care arm (*p* = 0.002 or *p* < 0.001, respectively; Table 2).

Ab testing was more often the reason for therapy change in the Ab testing arm than in the usual care arm (*p* < 0.0001; Table 2). Clinical composite (either MS clinical worsening or MRI changes) was the most frequent reason for therapy change in both arms and more frequently indicated in the Ab testing than in the usual care arm (*p* = 0.011; Table 2). Other reasons for therapy change (i.e., patient decision, AE, pregnancy, financial, and desire to switch therapy) were infrequently reported in the study but were reported more frequently in the Ab testing arm than in the usual care arm (*p* < 0.011; Table 2).

### Exploratory outcome measures

#### Relationship between BAb and NAb status

The BAb and NAb status for the mITT population (both patient groups) with both initial and final assessments are reported in Table 3. The majority of patients who were BAb(−) at study initiation remained BAb(−) at the final assessment (570/624; 91%), while a quarter of patients (104/421; 24.7%) who were BAb(+) at the initial assessment were BAb(−) at the final assessment.

**Table 3:** BAb/NAb status in patients with both initial and last assessments

	**Status at last assessment, n (%)**			
**Status at initial assessment**	**BAb(−)**	**BAb(+)/NAb(−)**	**BAb(+)/NAb(+) moderate**	**BAb(+)/NAb(+) high**
BAb(−), n = 624	570 (91.4)^*^	40 (6.4)^‡^	7 (1.1)^‡^	7 (1.1)^‡^
BAb(+)/NAb(−), n = 222	81 (36.5)^†^	122 (55.0)*	16 (7.2)^‡^	3 (1.4)^‡^
BAb(+)/NAb(+) moderate, n = 69	14 (20.3)^†^	14 (20.3)^†^	29 (42.0)*	12 (17.4)^‡^
BAb(+)/NAb(+) high, n = 130	9 (6.9)^†^	2 (1.5)^†^	9 (6.9)^†^	110 (84.6)*

*Patients who maintained titre.

†Decrease in titre.

‡Increase in titre.

BAb(−), serum binding antibody negative; NAb(−), serum neutralizing antibody negative.

NOTE: BAb(−), <4 units; BAb(+), >4 units; NAb(−), <20 units; NAb(+) moderate, ≥20 units to <100 units; NAb(+) high, ≥100 units.

BAb, serum binding antibodies; NAb, serum neutralizing antibodies.

BAb and NAb titre levels fluctuated for approximately 50% of patients who were NAb(+) with moderate titres. More than 90% of NAb(+) patients who had high titres (≥100 units) at study initiation were NAb(+) at the last assessment (Table 3).

**Figure 2:**
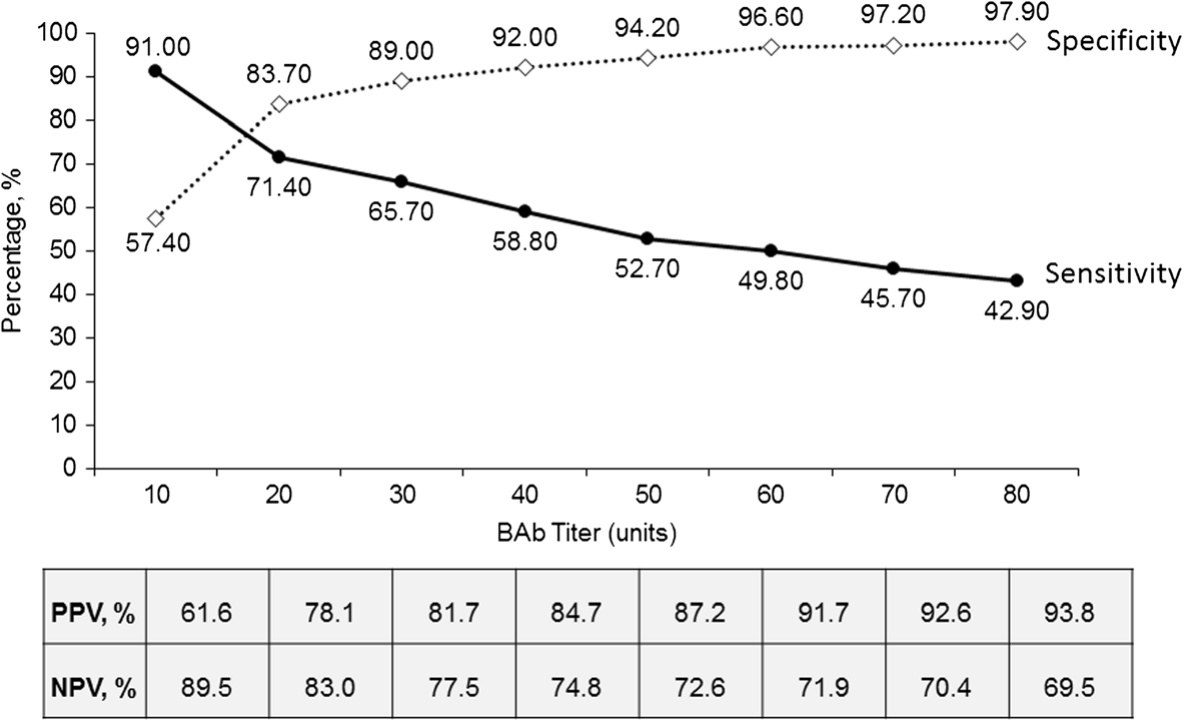
**Receiver operating characteristic curve and test characteristics for selected serum binding antibody (BAb) titres of predicting serum neutralizing antibody (NAb) positivity.** Positive predicted value (PPV) is the probability that the patient is truly NAb-positive (NAb >20), given that the patient has a positive test based on BAb titre (BAb > chosen threshold). Negative predicted value (NPV) is the probability that the patient is truly NAb-negative (NAb <20), given that the patient has a negative test based on BAb titre (BAb < chosen threshold).

AUC by ROC analysis was 0.86, indicating a strong correlation between BAb level and NAb(−) and NAb(+) status. Logistic regression analysis demonstrated BAb titres were a significant predictor of NAb positivity (*p* = 0.0012), with an odds ratio of 1.03 (95% confidence interval: 1.01 to 1.04). ROC analysis with test characteristics for selected BAb titres is illustrated in Figure 2. For example, when BAb = 20 units, the probability that the patient is truly NAb(+) is 78.1%.

**Figure 3:**
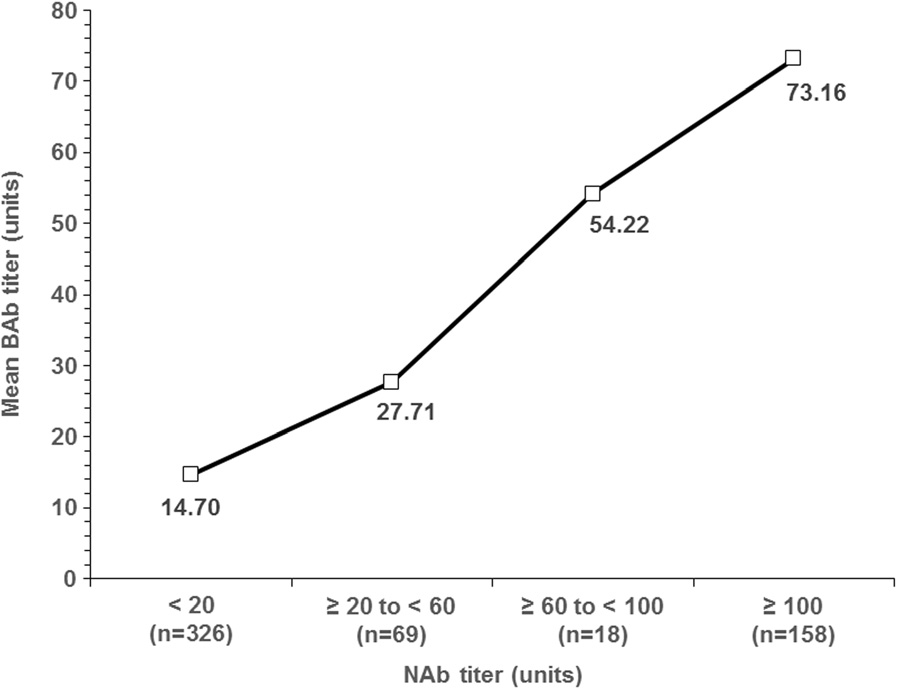
**Summary of the relationship between serum binding antibody (BAb) and serum neutralising antibody (NAb).** Mean (± standard deviation) BAb titre at various levels of NAb titre. τ = 0 · 54, *p* < 0.0001 for Kendall’s tau. R^2^ = 0.5264, *p* < 0.0001 using standard regression analysis.

An increased mean BAb level was observed with higher NAb titre categories. The correlation between BAb and NAb test results in the mITT population was statistically significant (*p* < 0.0001) using both Kendall’s tau (τ = 0.54) and standard linear regression (R^2^ = 0.5264), indicating a significant positive relationship between BAb and NAb test results (Figure 3).

#### Relationship between initial BAb and NAb status and type of/reason for therapy change

Antibody status and type of or reason for therapy change for the Ab testing and usual care arms are shown in Table 4. In the Ab testing arm, patients with an initial status of NAb(+) or BAb(+) were significantly more likely to change therapy (*p* < 0.0001 and *p* = 0.0054, respectively). Glatiramer acetate (*p* < 0.0001 for both) and closer vigilance were the most frequent types of therapy change in these patients (*p* < 0.0001 and *p* = 0.0004, respectively) (Table 4).

**Table 4:** Relationship between initial antibody status and types of and reasons for therapy change for the scheduled antibody testing arm and the usual care arm

**Outcome**			**Antibody status**					
			**BAb(−)**	**BAb(+)**	***p*-value**	**NAb(−)**	**NAb(+)**	***p*-value**
**Scheduled Ab testing arm, n**			**387**	**266**		**148**	**118**	
	Patients who changed therapy, n (%)*		62 (16.0)	66 (24.8)	0.0054	18 (12.2)	48 (40.7)	<0.0001
	Type of therapy change^†^							
		Start glatiramer acetate	10 (2.6)	37 (13.9)	<0.0001	6 (4.1)	31 (26.3)	<0.0001
		New/change in symptomatic therapy	10 (2.6)	10 (3.8)	0.3917	2 (1.4)	8 (6.8)	0.0096
		Closer vigilance	13 (3.4)	27 (10.2)	0.0004	8 (5.4)	19 (16.1)	<0.0001
	Reason for change*^,†^							
		NAb result	2 (0.5)	43 (16.2)	<0.0001	1 (0.7)	42 (35.6)	<0.0001
		MS clinical worsening	55 (14.2)	56 (21.1)	0.0222	26 (17.6)	30 (25.4)	0.0071
		MRI changes	11 (2.8)	22 (8.3)	0.0019	6 (4.1)	16 (13.6)	<0.0001
		Clinical composite^‡^	60 (15.5)	66 (24.8)	0.0031	27 (18.2)	39 (33.1)	<0.0001
**Usual care arm, n**			**343**	**233**		**119**	**114**	
	Patients who changed therapy, n (%)*		39 (11.4)	42 (18.0)	0.0241	24 (20.2)	18 (15.8)	0.5537
	Type of therapy change^†^							
		Start glatiramer acetate	4 (1.2)	13 (5.6)	0.0021	6 (5.0)	7 (6.1)	0.0247
		New/change in symptomatic therapy	8 (2.3)	5 (2.2)	0.8825	4 (3.4)	1 (0.9)	0.2681
		Closer vigilance	14 (4.1)	11 (4.7)	0.7117	5 (4.2)	6 (5.3)	0.5892
	Reason for change*^,†^							
		NAb result	1 (0.3)	2 (0.9)	0.5687	0 (0.0)	2 (1.8)	0.1015
		MS clinical worsening	32 (9.3)	41 (17.6)	0.0034	21 (17.6)	20 (17.5)	0.0809
		MRI changes	13 (3.8)	10 (4.3)	0.7628	6 (5.0)	4 (3.5)	0.7681
		Clinical composite^‡^	36 (10.5)	44 (18.9)	0.0043	23 (19.3)	21 (18.4)	0.1182

*Percentage based on total number of patients in category.

^†^Types of therapy change that were not significantly different for either arm included: current interferon beta (IFNβ), increase IFNβ, decrease IFNβ, start new IFNβ, start other immunotherapy, and initiate corticosteroids for relapse. Reasons for therapy change that were not significantly different for either arm included side effect, other concurrent illness/adverse event, laboratory abnormality, administrative/logistical reason, and other.

^**‡**^Either clinical worsening or MRI change.

Ab, antibody; BAb, serum binding antibody; MRI, magnetic resonance imaging; MS, multiple sclerosis; NAb, serum neutralizing antibody.

Reasons for therapy change for patients with positive Ab status within the Ab testing arm include NAb results (*p* < 0.0001 for both arms), MS clinical worsening (*p* = 0.0071 and *p* = 0.0222 for NAb[+] and BAb[+], respectively), MRI changes (*p* < 0.0001 and *p* = 0 · 0019, respectively), or clinical composite (*p* < 0.0001 and *p* = 0.0031, respectively; Table 4).

In the usual care arm, initial BAb(+) status had a significant effect on the overall number of patients who had a change in therapy (*p* = 0.0241; Table 4). A greater proportion of patients with initial BAb(+) versus BAb(−) status cited clinical worsening of MS (*p* = 0.0034) or clinical composite score (*p* = 0.0043) as their reason for therapy change. BAb(+) patients receiving usual care had more clinical relapses but not more MRI activity compared with BAb(−) patients receiving usual care, while BAb(+) patients had both more MRI activity and clinical relapses than BAb(−) patients when they received scheduled testing. NAb(+) patients had significantly more MRI activity (*p* = 0.0001) and clinical relapses (*p* = 0.0071) compared with NAb(−) patients when they received scheduled testing, but this was not observed when they received usual care (Table 4).

### Targeted adverse events

When analyzing both arms together, patients with NAb titres ≥100 units were significantly less likely to have flu-like symptoms (*p* < 0.001), injection-site reactions (*p* < 0.001), and depression (*p* < 0.046) compared with BAb(−) patients (Table 5). Moreover, patients with an NAb titre of 20 to 100 units were significantly less likely to have flu-like symptoms (*p* < 0.005) or injection-site reactions (*p* = 0.003) compared with BAb(−) patients (Table 5). The individual treatment arms had similar findings (data not shown).

**Table 5:** Relationship between antibody level throughout the study and targeted side effects for both arms combined (mITT cohort) and individual arms*

**Category**	**Antibody level (units)**	**Odds ratio**	**95% confidence interval**	***p*-value (odds ratio)**	***p*-value(linear trend)**
**Targeted side effects for both arms combined (mITT cohort)**					
Flu-like symptoms in past month	NAb ≥100	0.33	(0.22, 0.48)	<0.001	<0.001
	NAb 20 to <100	0.41	(0.28, 0.60)	<0.005	
	NAb <20	0.92	(0.71, 1.18)	0.50	
	BAb(−)	(Ref)	—	—	
Injection site reactions in past month	NAb ≥100	0.34	(0.25, 0.45)	<0.001	<0.001
	NAb 20 to <100	0.59	(0.41, 0.83)	0.003	
	NAb <20	0.79	(0.62, 1.01)	0.062	
	BAb(−)	(Ref)	—	—	
Depression in past month	NAb ≥100	0.70	(0.50, 0.99)	0.046	0.083
	NAb 20 to <100	0.99	(0.68, 1.44)	0.95	
	NAb <20	0.93	(0.71, 1.22)	0.60	
	BAb(−)	(Ref)	—	—	

*Repeated measures multivariable model utilized all data for which BAb/NAb testing and adverse events were assessed at the same visit. Models were adjusted for age, sex, study arm, multiple sclerosis type, current interferon, time from onset of symptoms, and centre size. Patients who were BAb(−) served as the referent, with an odds ratio calculated to represent the odds that a particular NAb level group would experience the event, compared with the referent. The linear trend *p*-value tested for the presence of a linear trend across BAb/NAb groups. Targeted events that did not show a statistically significant effect are not included in the table (seizures, menstrual irregularities, abnormal liver function tests, cytopenia, and decreased hemoglobin).

BAb, serum binding antibody; mITT, modified intention-to-treat; NAb, serum neutralizing antibody.

There was a significant inverse linear correlation between NAb titre level and flu-like symptoms (*p* < 0.001) and injection-site reactions (*p* < 0.001) (Table 5). The findings for both arms combined (mITT cohort) were similar to the individual treatment arms, except that there was a trend for depression associated with NAb titre level when both arms were combined.

## Discussion

In the present study, knowledge of Ab test results had an impact on therapy or management choices. More patients in the Ab testing arm had therapy changes than in the usual care arm during the 12-month follow-up. It is important to note that this was a relatively large study with a high rate of completion. Further, all time points were analyzed in both arms, and the endpoint of change in therapy by the 12-month time point was statistically significant. Thus, it is unlikely that the mITT criteria for the different patient cohorts would have had a significant effect on the results presented.

Our findings are consistent with those of a recently published study conducted to determine whether early BAb titres could predict NAb development. In that study 78.9% of 164 patients receiving de novo IFN-β treatment were BAb(+) after 3 months. The investigators found that BAb titres ≥ 1:2400 at 3 months predicted NAb evolution with a sensitivity of 74.7% and a specificity of 98.5% [14].

Consistent with previous study findings [1], our study found NAb positivity to vary by formulation and dosage frequency of IFNβ treatment. Patients who received IFNβ-1a 44 μg subcutaneously t.i.w. had higher positive NAb test results than patients on IFNβ-1b 250 μg on alternate days or IFNβ-1a 22 t.i.w. This finding suggests that consideration of the rate at which Ab titres develop for different treatment regimens is warranted.

As with NAb(+) status, BAb(+) status was shown to predict treatment patterns. An initial BAb(+) status had a significant effect on the overall number of patients changing therapy and on clinical manifestations of MS. The frequency and risk of AEs typically associated with IFN therapy were lower in subjects with higher NAb titres, which suggests that the neutralising effect of the antibodies on IFNβ may have caused a reduction in these events. This association between NAb titre and AEs is supported by previous reports.

Overall, data support the use of BAb titres from Ab testing to predict NAb positivity. We found that if a patient had BAb >50 units, there was a very high probability that the patient was NAb(+), and treatment decisions could be adequately based on this information.

Although there are no widely accepted guidelines on BAb test-driven management, a titre cutoff point for BAb could be used to predict the risk of NAb(+) and the necessity for medication change [15]. A recent study [16] suggested using a high BAb titre as a cutoff point for IFN therapy, because of a strong correlation between NAb and BAb in this group. Moreover, in patients with low titres, the study suggested refining the BAb assay by incorporating an MxA induction assay to establish whether the bioavailability of IFNβ is preserved. Results from our ROC analysis indicate there is a very high probability of patients with BAb >50 units (specificity ≥94%) and even as low as 20 units (specificity >80%) being NAb(+), which could mean that lower BAb titre cutoff points may be sufficient to guide treatment decisions.

### Limitations of study

One shortcoming of this study is the absence of paired clinical response data. Although the study was not designed to follow this outcome, the correlation between clinical worsening and NAb(+) response in patients on IFNβ therapy is well established [4,17]. Another consideration when interpreting the results is the imbalance in clinic visits that the Ab testing arm would have received versus the usual treatment arm, which could have affected management choices irrespective of Ab results. Moreover, it could be argued that the study duration was inadequate. However, the study duration of 12 months represents real-world practice and was sufficient to confirm both the relative stability of NAb/BAb levels and the strong correlation of BAb titres with NAb positivity. In addition, patients were required to complete 1–4 years of therapy before study entry, the time frame of greatest importance in NAb development.

It should be noted that our study involved formulations of IFNβ-1a currently available in the US, as used in the EVIDENCE and REGARD studies, Rebif^®^, 22 or 44 μg t.i.w., and Avonex^®^, 30 μg weekly (considered low dose); a newer formulation of Rebif^®^ has since been introduced in other countries with the aim of inducing less NAb positivity [18].

## Conclusion

Testing for NAb is an important aspect of MS management in terms of predicting treatment response to IFNβ. Using BAb testing before screening for NAb titres, and defining a cutoff point for the BAb titres at which to discontinue IFNβ therapy, may reduce the necessity for the more expensive NAb testing assays.

## Abbreviations

Ab: Antibody; AEs: Adverse events; AUC: Area under the curve; BAb: Serum binding antibodies; EFNS: European Federation of Neurologic Societies; IFNβ: Interferon-β; IgG: Immunoglobulin G; mITT: Modified intention-to-treat; MRI: Magnetic resonance imaging; MS: Multiple sclerosis; NAb: Neutralizing antibodies; ROC: Receiver-operating characteristic; t.i.w: Three-times weekly.

## Competing interests

EF has been a consultant for Acorda Pharmaceuticals, Bayer, Biogen Idec, EMD Serono, Genzyme, Novartis, Opexa, Sanofi-Aventis, and Teva Pharmaceuticals. EF has also been a clinical investigator for the following: Avanir, Biogen Idec, EMD Serono, GlaxoSmithKline, Teva Pharmaceuticals, Novartis, Ono, Sanofi-Aventis, Eli Lilly, and Roche Genentech. BG has received honoraria for speaking from Bayer, Biogen Idec, Genzyme-Sanofi, Novartis, and Teva Pharmaceuticals. CM reports consultancy/lectureship with Bayer, Biogen Idec, EMD Serono, Novartis, Teva Pharmaceuticals, and Wyeth Pharmaceuticals. CM has received grants from Bayer, Biogen Idec, EMD Serono, Novartis, and Teva Pharmaceuticals. RM has received honoraria for consulting, speaking, and teaching from Teva Pharmaceuticals, Biogen Idec, Genzyme, and Acorda Therapeutics. RM has also received grant and research support from Teva Pharmaceutials, Biogen Idec, Novartis, and Acorda Therapeutics. RM participated on advisory committees or review panels for Teva Pharmaceuticals, Biogen Idec, Novartis, and Genzyme. ADG has received honoraria for consulting services from Acorda Therapeutics, Avanir, Biogen Idec, EMD Serono, Genzyme-Sanofi, Novartis, Pfizer, and Teva Pharmaceuticals and financial support for research activities from Acorda Therapeutics, Biogen Idec, EMD Serono, Genzyme, Novartis, Ono, Sun Pharma, Takeda, and Teva Pharmaceuticals. SJG, PL, and JNC are employees of Teva Pharmaceutical Industries, Kansas City, MO, USA.

## Authors’ contributions

All authors contributed equally to the development of this manuscript. All authors read and approved the final manuscript.

## Pre-publication history

The pre-publication history for this paper can be accessed here:

http://www.biomedcentral.com/1471-2377/14/73/prepub
